# Repeated testing improves achievement in a blended learning approach for risk competence training of medical students: results of a randomized controlled trial

**DOI:** 10.1186/s12909-017-1016-y

**Published:** 2017-09-26

**Authors:** C. Spreckelsen, J. Juenger

**Affiliations:** 1 Department of Medical Informatics, Medical Faculty, RWTH Aachen University, Pauwelsstr. 30, 52074 Aachen, Germany; 2 Department of Psychosomatic and General Internal Medicine, University of Heidelberg, Im Neuenheimer Feld 410, Heidelberg, 69120 Germany; 3 Institute for Medical and Pharmaceutical Tests, Große Langgasse 8, Mainz, 55116 Germany

## Abstract

**Background:**

Adequate estimation and communication of risks is a critical competence of physicians. Due to an evident lack of these competences, effective training addressing risk competence during medical education is needed. Test-enhanced learning has been shown to produce marked effects on achievements. This study aimed to investigate the effect of repeated tests implemented on top of a blended learning program for risk competence.

**Methods:**

We introduced a blended-learning curriculum for risk estimation and risk communication based on a set of operationalized learning objectives, which was integrated into a mandatory course “Evidence-based Medicine” for third-year students. A randomized controlled trial addressed the effect of repeated testing on achievement as measured by the students’ pre- and post-training score (nine multiple-choice items). Basic numeracy and statistical literacy were assessed at baseline. Analysis relied on descriptive statistics (histograms, box plots, scatter plots, and summary of descriptive measures), bootstrapped confidence intervals, analysis of covariance (ANCOVA), and effect sizes (Cohen’s d, r) based on adjusted means and standard deviations.

**Results:**

All of the 114 students enrolled in the course consented to take part in the study and were assigned to either the intervention or control group (both: *n* = 57) by balanced randomization. Five participants dropped out due to non-compliance (control: 4, intervention: 1). Both groups profited considerably from the program in general (Cohen’s d for overall pre vs. post scores: 2.61). Repeated testing yielded an additional positive effect: while the covariate (baseline score) exhibits no relation to the post-intervention score, F(1, 106) = 2.88, *p* > .05, there was a significant effect of the intervention (repeated tests scenario) on learning achievement, F(1106) = 12.72, *p* < .05, d = .94, *r* = .42 (95% CI: [.26, .57]). However, in the subgroup of participants with a high initial numeracy score no similar effect could be observed.

**Conclusion:**

Dedicated training can improve relevant components of risk competence of medical students. An already promising overall effect of the blended learning approach can be improved significantly by implementing a test-enhanced learning design, namely repeated testing. As students with a high initial numeracy score did not profit equally from repeated testing, target-group specific opt-out may be offered.

## Background

Tests can force learners to activate knowledge or skills. This activation impacts learning: Test-enhanced learning has been shown to have marked effects on achievement and retention [1]. Test-enhanced leaning could be used in medical education to effectively improve the training of competences, which are still underrepresented, despite their relevance. Diagnostic advice and therapeutic decisions rely on good risk estimation and risk communication (RE&C) skills. Nonetheless, there is striking evidence of a marked lack of RE&C skills in many physicians [2]. RE&C learning objectives are underrepresented in medical curricula. Therefore, effective and sustainable training approaches are required. Consequently, it is promising to adopt test-enhanced learning to curricular training of RE&C skills.

Test-enhanced learning was defined as “increased retention of knowledge or skills that is produced by the act of retrieval during testing” [3]. Justifications of test-enhanced learning explain its effect by two main factors: first, the enhanced exposure to learning content (“total-time hypothesis”) [4, 5] and, second, the stimulation of cognitive effort due to recalling knowledge in order to solve the test (“retrieval hypothesis”) [6]. Dedicated instructional designs implement test-enhanced learning by repeated testing and by feedback leading to self-regulation [7]. Repeated testing can be further improved by spaced learning. Spaced learning fosters retention effects by a careful adjustment (increasing) of time-intervals between test repetitions [6, 8]. Studies in a laboratory setting suggested that repeated testing has a strong effect on long-term retention and improves the ability to allocate cognitive effort effectively (meta-level learning) [6, 9]. Test-enhanced learning was successfully established in medical education on graduate and postgraduate level, in dental education, and in nursing education [10–13]. Test-enhanced learning was investigated in the context of clinical reasoning and human error prevention [14, 15], but so far no study addressed repeated testing in the context of RE&C training.

Test-enhanced learning may profit largely from blended learning (BL). BL combines “different modes of delivery, models of teaching and styles of learning” [16]. Driven by technology, many BL approaches focus on complementing face-to-face teaching with computer/web based learning in order to combine their respective strengths. Many learning management systems support online tests with learner feedback. Therefore, blended test-enhanced learning can be implemented efficiently by combining face-to-face teaching, online delivery of learning content, and online self-tests in a BL scenario.

Statistical literacy is a necessary condition for avoiding critical misinterpretations of statistical data, clinical studies and quantitative tests. The term was coined by Gigerenzer et al. [17], who urged dedicated effort to improve the risk estimation and communication skills (RE&C) of physicians. Different studies showed consistently that many physicians fail in interpreting screening statistics and cannot estimate even roughly the predictive values given the sensitivity and specificity of a diagnostic test [2, 18, 19]. A lack of statistical literacy jeopardizes adequate consultation, informed consent, and shared decision making [18, 20, 21]. As an example, an obvious lack of RE&C competence is held responsible for suicides induced by misinterpreted HIV-test results: As doctors assumed that high test sensitivity always implies equally high predictive values they (incorrectly) told test-positive patient they were almost certainly infected [17]. A recent study yielded that many counselors still communicate utterly incorrect interpretations of HIV test performance to their clients [22]. RE&C skills, therefore, need to be addressed systematically and effectively by medical curricula. Training of RE&C skills can profit from given evidence: Studies yielded, that instructing medical students to use natural frequencies as a representational means for probabilities led to marked and sustainable achievements in RE&C skills [23]. Recently, Caverly et al. [24] developed an instrument for assessing skills in critical risks interpretation. In addition, the choice of adequate means for communicating risks/chances to patients has been thoroughly investigated in the recent years yielding evidence of 1) marked effects and 2) reasons for preferring specific formats of communication [25–27]. Medical education can, therefore, use these results in order to specify adequate learning objectives for RE&C training.

Despite the need for RE&C training, no dedicated RE&C program regularly integrated in a medical curriculum has been reported. Furthermore, no study has yet addressed the effect of test-enhanced learning in curricular RE&C training for medical students. Blended, test-enhanced learning seems an effective approach to curricular RE&C training. As online tests can be easily delivered in a BL scenario, repeated testing can be considered a particularly suitable form of blended, test-enhanced learning. In view of the opportunities of applying repeated testing to blended RE&C training of medical students, a thorough investigation of its impact on learning achievement was needed. Our study aimed to investigate this impact by a randomized controlled trial.

## Methods

### Cohort recruitment and flow of the study

Participants were recruited from students of the Aachen medical school enrolled in the mandatory course “Evidence-based Medicine” (EBM). The learning objectives of the EBM course include a subset of RE&C related topics. For organizational reasons the complete year of medical students is regularly split in two sub-cohorts, which attend the course subsequently due to course rotation. The study included the complete course cohort of the summer term 2015 and, therefore, represented a typical cross-section of medical students in the third year of our medical curriculum.

The repeated testing scenario was piloted in the term preceding the study (winter term 2014/15) in order to test the reliability of the technical platform, student compliance, and the comprehensibility of the self-test items. No control group was established; all students enrolled in the EBM course were included in repeated testing.

**Fig. 1:**
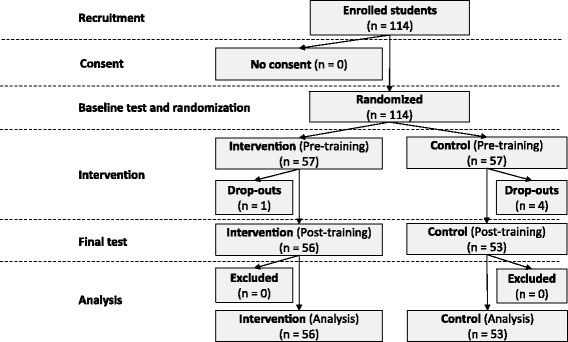
Intervention integrated in the previously existing course “Evidence-based Medicine” (EBM)

In the following term the complete course cohort of the EBM course was invited to take part in the study and was asked for consent. The participants filled out a profiling questionnaire (see below) and answered the baseline test. Subsequently, the participants were randomly assigned to the intervention group and the control group. After the EBM course, i.e. 9 weeks after the start of the study, post-testing took place. Figure 1 shows the flow of the study.

### Sample size calculation

Sample size estimation relied on Borm’s approach [28, 29]: The calculation found that *n* = 46 was needed for a power of .8 at significance level α = .05 if a middle-sized effect (d = .4) and correlation (R-squared = .7) were assumed.

### Randomization

Balanced randomization was carried out by ordering the list of participants by random numbers (generated by the R environment for Statistical Computing) and dividing the list equally.

### Intervention

This study introduced repeated testing of risk estimation/communication (RE&C) skills in the context of an existing BL module on EBM. The EBM course combined attendance teaching (lectures), training in the computer lab, online tutorials, and online tasks and tests. The control group as well as the intervention group followed this BL approach.

The primary intervention of the study was to let students work on a sequence of six short online-tests (10–15 min, one test per week), which provided RE&C related items. The sequence implemented a repeated testing approach, based on multiple choice items and items asking for numerical input. The students received electronic feedback on their test-performance immediately after submitting the completed test. All students were informed that the results of the tests would not influence their grades. They were also informed that completion of all tests was required for admittance to the final course exam. The online-tests (and additional learning material) were delivered by the Learning Management System (LMS) Moodle (https://moodle.org/). The LMS logged the students’ activities, which enabled assessing of their compliance with the online-tests afterwards. The tests were accessible only after individual login. Each test was only available for a predefined interval of 6 days after each lecture, which enabled spaced learning as specified in the introduction. The LMS allowed to check for compliance and to send a reminder to students, who failed to complete the test in time. In these cases the deadline was extended by 5 days.

Both, the intervention group and the control group, had to work equally on short online tasks each week. The only difference between intervention and control was the presence vs. absence of repeated online tests concerning RE&C competencies. In the case of the control group these tests were substituted by other online tasks.

**Fig. 2:**
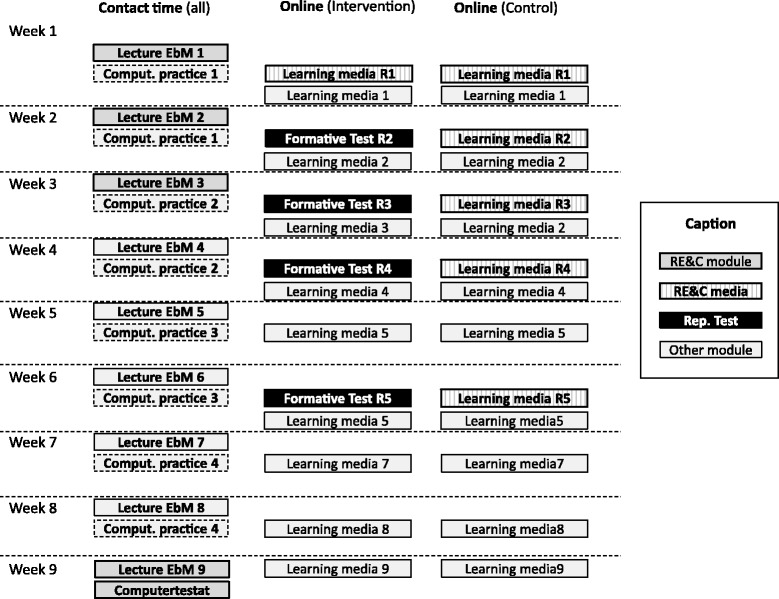
Flow of participants

Figure 2 gives an overview of the EBM module, which includes nine lectures (90 min) and four units of computer practice (90 min per student) of contact time and additional online training. Parts of the online training were always available to the students. Other parts were strictly scheduled and, therefore, enabled spaced and repeated testing. Differences between the test and control group in the course of the existing EBM module are highlighted in Fig. 2.

### Outcome and measurement

The primary endpoint (dependent variable) of this study was the participants’ learning achievement concerning a set of operationalized learning objectives RE&C. Learning achievement was measured by the difference in the scores of a pre- and post-training test. Details of the test instrument are given below.

The primary independent variable was the participants’ assignment to either the control or intervention group.

The study used two different instruments for data acquisition: a short profiling test (five items) and a test of RE&C skills (nine items). The profiling test was used only at baseline, whereas the nine RC&E items formed the pre- and post-training test.

The first three items of the profiling test represent the Basic Numeracy Test introduced by Schwartz et al. [30] and used previously in studies concerning statistical literacy. The fourth profiling item addressed the concept of statistical independence using roulette as an example. The last profiling item asked the students to decide, which of five given methods yield 100% correct results, thereby investigating the students’ awareness of the general lack of absolute certainty. The total number of correct answers to the five profiling items served as a score (profiling score) with values ranging between zero and five.

The nine items on RE&C skills were inspired by the aspects of basic statistical literacy introduced by Gigerenzer et al. [17]. Table 1 gives an overview of the items and the learning objectives addressed.

**Table 1:** Overview of the items used for profiling basic numeracy and awareness of probability (profiling items) and the items addressing risk estimation and communication (RE&C items) ^a^

Item	Topic	Objective	Type
P1	Transform a percentage into an integer value	(Profiling participants’ basic numeracy)	Num
P2	Transform integer values into percentages	(Profiling participants’ basic numeracy)	Num
P3	Give expectation for the result of the next coin flip in a sequence of coin flips	(Profiling participants’ concept of probability)	MC
P4	Estimate the chance of the next color being black in roulette	(Profiling participants’ concept of probability)	BA
P5	Rate certainty of detection methods (e.g. certainty of a DNA-test)	(Profiling participants’ awareness of uncertainty)	BA
RE&C items		Objective	Type
I1	Define five-year mortality (“In the context of a study on the mortality of a disease you are going to investigate the five-year mortality. When does the five-years interval start?”)	Interpret and explain five-year mortality	MC
I2	Substantiate dual formulation for risk communication (“For communicating risks to patients it is recommended to adopt “dual wording”. Which effect should be avoided by this measure?”)	Explain framing effect and its consequences	MC
I3	Interpret specificity of diagnostic test (“In court proceedings concerning medical malpractice it is discussed, which diagnostic tests should be accepted. Which feature would make a test unfavorable to a defendant hospital, because the test is more likely to indicate a non-existing complication than other tests?”)	Interpret and explain test characteristics	MC
I4	Communicate the risk of being ill, given a positive test result (“You intend to tell a patient, how likely it is to actually have a certain disease, in case that the test yields a positive result. Which of the following five formulations is NOT suitable here?”)	Communicate risks adequately	MC
I5	Compare absolute vs. relative risk (“You have to make a therapeutic decision. By literature research you find two relevant RCTs. The first study found out that therapy A leads to a relative risk reduction of 0.1%. The second yielded an absolute risk reduction of 1%. Which of the following statements is correct?”)	Interpret risks adequately	MC
I6	Interpret five-year mortality (screening context) (“The implementation of a screening program increased five-year mortality from 30% to 100%. Clinical Studies found out, that, nonetheless, there is no difference in mean life expectancy between patients included in the screening program and patients not included. What is the reason behind this discrepancy?”)	Explain benefits and harms of interventions	MC
I7	Calculate true positives from prevalence and sensitivity (“A given disease has 1% prevalence in standard population. The sensitivity of a diagnostic test is 85%. In how many cases out of 10,000 the test correctly indicates the disease?”)	Interpret and explain test characteristics	MC
I8	Calculate absolute risk given mortality of test vs. control group (“Experts discuss the implementation of a screening program, which examines the participants every three years over an interval of 10 years. Participating in the program reduces the relative risk to die from the disease by 50%. Without screening, four out of 1000 patients die from the disease. What is the correct absolute risk reduction here?”)	Interpret and communicate risks adequately	MC
I9	Calculate predictive values given prevalence and test characteristics (“Trisomy 21 is present in 1% of pregnancies of 40 year old women. Non-invasive tests have a sensitivity of about 90% and a specifity of about 95%. What is the probability of a trisomy 21 in case of a positive test result?”)	Understand and communicate test results	Num

^a^Item types: *BA* binary alternative (Y/N), *MC* multiple choice, *Num* numerical input

Except for one item expecting a numerical answer, all items were multiple choice questions (Type A: single positive choice out of five possible answers). Four items required the students to calculate numbers from given values before answering the question (e.g. values for prevalence, mortality, sensitivity, specifity). In these cases the values used in the final exam differed from the values used in the baseline test to prevent the students from reusing numerical results learned by heart. Apart from that, the wording of the items was kept exactly the same.

As mentioned above: all nine items of the baseline RE&C test (pre-training test) were repeated in the final exam (post-training test). The total number of correct answers to the nine RE&C items served as pre-training score (baseline) and post-training score, respectively, with values between zero and nine.

### Data analysis

To be able to associate the individual baseline score to the corresponding score of the final exam the baseline data were pseudonymized by applying the secure hash algorithm (SHA-1) to the students’ matriculation numbers. After combining the baseline data and the final assessment, the joined dataset was completely anonymized before being further processed by statistical analysis.

Histograms and a summary of descriptive statistics were used to compare the characteristics of the intervention group and the control group. In order to examine the primary endpoint of the study 95% confidence intervals for the mean scores of the pre- and post-test were calculated by non-parametric bootstrapping and plotted as a line chart. Non-parametric bootstrapping constructs confidence intervals by resampling (randomly drawing sub-samples with replacement) from the observed data [31]. Internal consistency reliability of the test was calculated using Cronbach’s alpha.

Subsequently, an analysis of covariance (ANCOVA, Type III sums of squares) served as a means for testing the initial hypothesis. The ANCOVA used the group as the independent variable and the final assessment score as the dependent variable while treating the baseline score as a covariate. Effect sizes (Cohen’s d, r) were calculated using adjusted means and standard deviations, respectively.

Statistical analysis used the R environment (The R Project for Statistical Computing, https://www.r-project.org/). For nonparametric bootstrapping the R-package Hmisc [32] was adopted using the default of 1000 bootstrap resamples. The R-package Psych [33] was used for calculating Cronbach’s alpha.

The profiling score (ranging between zero and five) was used to compare participants with a low profiling score to those with a high score. Originally, we planned to define the high performing subgroup by the third quartile of the distribution of profiling scores. After data acquisition it turned out, that this approach was not feasible (see the results section): Most of the participants in both groups achieved a profiling score of four. Therefore, this value represented median, first, and third quartile of the distribution. As a consequence, we had to define high performers in a different way and decided to compare the participants with a score ranging between zero and four (i.e. a profiling score ≤ 80%) to those, who achieved a score of five (100%). This was the least arbitrary choice and roughly approximates the top- 25% of performers. We compared the difference between pre- and post-training RE&C scores of the high profiling scores subgroup (profiling score > 80%) to that observed in the other subgroup (profiling score ≤ 80%). Again we used confidence intervals calculated by non-parametric bootstrapping and line charts.

## Results

The repeated testing scenario was piloted in the winter term preceding the actual study (2014/15). All students enrolled in the EBM course (*N* = 150) participated in the pilot intervention; no control group was established. The pilot confirmed the technical reliability of the LMS. There was no critical failure during the course. Five students failed to submit two out of six self-tests, and 16 students failed to submit one self-test in time (yielding a total rate of 14% non-compliants). Compliance decreased over time: in the case of test 1 there was only one non-compliant, while there were 3, 2, 4, 4, and 13 for tests 2–6, respectively. As a result of distractor analysis one test-item (concerning the interpretation of predictive values) was reworded due to a random (i.e. approximately equal) distribution of answers over all answer options including the correct one.

The study took place during the summer term 2015 (April 7th to July 17th, 2015). Figure 1 shows the resulting flow of participants: All 114 students enrolled in the EBM course consented to take part in the study and were assigned to either the intervention or control group (both: *n* = 57) by balanced randomization. The intervention group included 47 female and 10 male students; the average age was 21.7 (SD: 2.1). The control group included 44 female and 13 male students; here the average age was 22.2 (SD: 2.45). Thus, there were no marked differences between the groups concerning these features. Five participants dropped out due to non-compliance with the online activities of the blended learning course.Profiling scores (with possible values between zero and five) indicated very good performance: Only 18.87% and 14.28% of the control group and intervention group, respectively, had a score of less than 4 (i.e. fewer than 80% of the items), 58.49% and 64.29% had a score of exactly 4 (i.e. 80% of the items), while 22.64% and 21.42% earned the best possible score of 5. The distribution and cumulative frequencies of the profiling scores for intervention vs. control yielded no marked differences. The score of 4 (i.e. 80% of the items) represented the median, first, and third quartiles as well. As a consequence, comparison of low and top performers based on quartiles was not feasible. As argued in the methods section, choosing the participants with a profiling score above 80% was the least arbitrary choice and roughly approximated the top- 25% of performers. Thus, we used this criterion for subgroup definition.

With respect to pre-training test performance, i.e. the RE&C score, there were differences between the intervention group and control group. Table 2 gives an overview on the pre-training and post-training scores achieved by the intervention group and the control group, respectively. As already considered by choosing ANCOVA, these differences require control of the pretest performance when testing the study hypothesis.

**Table 2:** Overview of the RE&D Scores (before training, after training, and difference)

Group	Pre-training RE&D Score	Post-training RE&D Score	Difference
Intervention	3.61 (SD: 2.86)	8.18 (SD: 1.46)	4.57 (SD: 3.48)
Control	3.04 (SD: 1.88)	6.98 (SD: 3.71)	3.94 (SD: 4.82)

Based on the post-training test results, the calculation of Cronbach’s alpha was .71 indicating acceptable (but rather moderate) reliability based on internal consistency.

Independent from the intervention, all participants profited considerably from the program (Cohen’s d for overall pre vs. post scores: 2.61).

ANCOVA indicated that the covariate (pre-training score at baseline) was not related to the post-training score, F(1, 106) = 2.88, *p* > .05, while there was a significant impact of the intervention (repeated testing) on learning achievement, F(1106) = 12.72, *p* < .05, d = .94, *r* = .42 (95% CI: [.26, .57]). These results show that, even when controlling for the effect of the baseline (treated as a covariate), the post-training test score depends significantly on the group (intervention vs. control group). Thus, including repeated testing into an existing blended learning design led to significantly higher learning achievement, which cannot be explained otherwise i.e. by differences between prior knowledge/skills in the intervention group and the control group, respectively.

**Fig. 3:**
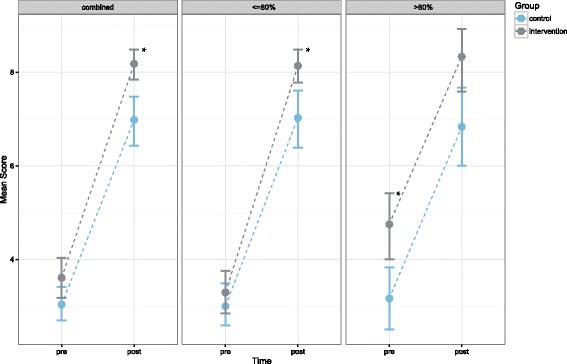
Mean scores of the pre- vs. post-training test for the intervention (blue) vs. control (grey). The three diagrams compare subgroups with a different score in the profiling test, which addressed basic numeracy plus the awareness of statistical independence and general uncertainty. The vertical lines show the 95% confidence intervals calculated by bootstrapping. The star symbol indicates significant differences between intervention and control group

Calculation of the adjusted means yielded 7.03 (95% CI: [6.60, 7.47]) and 8.13 (95% CI: [7.71, 8.56]) for the control and intervention group, respectively. Figure 3 shows means for pre- and post-intervention scores with bootstrapped 95% confidence intervals for all participants (combined) and for subgroups scoring high (score > 80%,) and low (≤80%) in the profile items concerning basic numeracy and statistical literacy, respectively.

## Discussion

This study showed that some relevant RE&C skills of medical students can be trained efficiently by a dedicated blended learning program integrated into the medical curriculum. With respect to the aim of the study our data show that the marked overall effect of the blended learning approach (as measured by RE&C related MC- and numerical items) can be improved significantly by implementing repeated testing on top of the blended learning approach. Students with high profiling score did not profit equally from repeated testing: In this group the study did not find a positive effect of repeated testing on top of the effect of the standard training.

For the complete cohort the results confirm our initial hypothesis: The ANCOVA results and the separate 95% confidence intervals of the mean scores at post-training time clearly show the effect of the intervention. The non-parametric bootstrapping procedure used for calculating the confidence intervals does not rely on specific assumptions concerning the shape of the underlying distribution, which strengthens the result. Repeated testing, therefore, can add significantly to the achievement of risk estimation and communication related learning goals in our BL scenario.

Obviously, the marked learning effect of the training module in general (pre-training vs. post-training) exceeds the additional effect of the repeated testing intervention.

Our instrument used to measure the learning outcome contained only multiple choice items and numerical items. Assessing risk communication in a realistic context (e.g. assessment based on standardized patients) was far beyond the scope of the study. Nonetheless, some of the RE&C items required the participants to calculate and estimate risks starting from information given in a typical clinical decision context. Consequently, these items covered the skills of risk estimations relevant to clinical situations. Validity was also fostered by deriving the items systematically from RE&C related learning objectives defined by the catalogue of operationalized learning objectives of our faculty.

The standard training in both groups yielded a high effect size. Compared to this effect, the additional effect of repeated testing was relatively small. Therefore, one could argue that the benefit of the intervention was too small to justify the additional effort required to implement the intervention or even the additional workload for the students. In contrast, both the implementing effort and the additional workload would be overrated, if only considered in a short-term perspective. Since the electronic self-assessment could be reused in future courses, the implementation costs per term decrease drastically. And, as suggested by informal feedback from the students, the additional workload induced by the repeated tests reduced the workload necessary for preparing for the final exam. As an additional effect, obligatory repeated tests included in the BL design and offered during short intervals forced the students to access the online training regularly. Repeated testing may, thus, foster adherence to blended learning in general.

The study showed that there was a significant effect of repeated testing on the outcome in general. Nonetheless, the additional investigation of subgroups of the initial profiling items raises doubts as to whether high performers of the profiling items profit from the intervention at all. Given the considerable workload of repeated testing, one could well argue, that repeated testing is unjustified in the case of these high performers. The matter might be solved by establishing the profiling test as a routine and then allowing high performers to opt out of repeated testing.

Cantillon [34] pointed out possible problems of test-enhanced learning in medical education and stressed that it “needs to be evaluated as a curriculum-wide strategy to avoid skewing learning study behaviour”. He questioned the compatibility of test-enhanced learning with multiple assessment formats used in the context of competency-based programs. In addition, he stressed the need to investigate the effect of improved retention on actual problem solving. These questions are still relevant and should be addressed by further research on the subject.

### Limitations

The study context was defined not only by the learning objectives and content of the RE&C training, but also by the BL design and environment. BL had specific implications for the experimental manipulation: The introduction of repeated tests might have been hampered or more costly, if no BL infrastructure had been available. Without the electronic BL environment it would have been much more difficult to track students’ adherence to the repeated test protocol. Timely individual feedback concerning adherence might also not have been feasible. In contrast, the BL environment allowed implementing repeated tests, instant feedback to students about test results, and tracking adherence easily and without unreasonable effort.

Blinding was not feasible due to the students’ different learning activities induced by the intervention. In order to keep the bias as low as possible, the repetitive self-tests were delivered automatically to the participants by the LMS, while nearly everything in the virtual course room looked the same for the intervention and control group.

Most items of our baseline-/post-training test address factual knowledge and, thus, learning objectives at a low level of competence. The items requiring calculation go beyond the (cognitive) level of factual knowledge. Nonetheless, one could still argue that risk estimation and communication skills on higher competence levels are not adequately addressed by this study. For instance, the training did not address the need of assessing the quality of information given by a patient (Did he/she lie?) or the ability to judge whether a patient actually understands the risk figures. Furthermore, the study design did not include long-term follow-up. An investigation into long-term retention was beyond the scope of the study. Obviously, these important aspects need to be addressed by future investigations.

## Conclusions

Including repeated online tests in a BL scenario can improve learning achievement in medical curricula. Our study showed that the training of RE&C competences profited considerably from this repeated testing. Nonetheless, this study did not address complex RE&C skills on higher competence level such as detecting false information deliberately given by patients. Thus, it is not clear so far, whether suitably designed repeated testing can support training in higher level RE&C skills as well. Students with high initial numeracy score did not profit equally from repeated testing in RE&C. As a consequence, opting out of repeated testing could be offered to this subgroup in the future with the provision of initial student profiling. Our results suggest promoting the use of repeated testing in order to foster fields still underrepresented in medical curricula despite their relevance.

## Abbreviations

ANCOVA: Analysis of Covariance CI Confidence Interval EBM Evidence-based medicine LMS Learning management system RE&C Risk estimation and communication SHA-1 Secure hash algorithm
